# LncRNA TUSC8 suppresses the proliferation and migration of esophageal cancer cells by downregulation of VEGFA

**DOI:** 10.7150/jca.57814

**Published:** 2021-09-03

**Authors:** Rui Hu, Rui Bi, Lianyong Jiang, Xuhui Yang, Yuan Zhong, Xiao Xie

**Affiliations:** Department of Cardiothoracic Surgery Xinhua Hospital Affiliated to Shanghai Jiaotong University School of Medicine, Shanghai 200092, China.

**Keywords:** LncRNA TUSC8, VEGFA, Esophageal cancer, Proliferation, Migration

## Abstract

**Objective:** This study aims to determine the expression pattern of long non-coding RNA (lncRNA) TUSC8 in esophageal cancer tissues and cell lines, to investigate its effects on esophageal cancer cell proliferation and migration, and to explore the mechanism of TUSC8-mediated esophageal cancer suppression via VEGFA downregulation.

**Patients and Methods:** TUSC8 levels in esophageal cancer tissues and cell lines were detected by quantitative real-time polymerase chain reaction (qRT-PCR). The influence of TUSC8 on clinical features in esophageal cancer patients was analyzed. After intervening TUSC8 expression in esophageal cancer cells, the proliferative and migratory abilities were examined in OE19 and TE-1 cells through a series of function experiments. The interaction between TUSC8 and VEGFA was assessed by the bioinformatics prediction and dual-luciferase reporter assay. Finally, the co-regulation of TUSC8 and VEGFA on esophageal cancer cell functions was evaluated.

**Results:** TUSC8 was downregulated in esophageal cancer tissues compared with normal ones. Identically, decreased TUSC8 expression was detected in esophageal cancer cell lines compared with control cells. Low TUSC8 expression predicted poor prognosis in patients with esophageal cancer. Knockdown of TUSC8 promoted the proliferative and migratory abilities in OE19 cells, whereas overexpression of TUSC8 resulted in opposite results in TE-1 cells. VEGFA was confirmed to be a target gene of TUSC8. Overexpression of VEGFA could reverse the regulatory effects of TUSC8 on esophageal cancer cell proliferation and migration.

**Conclusions:** LncRNA TUSC8 is downregulated in esophageal cancer tissues and cell lines. TUSC8 inhibits the proliferative and migratory abilities in esophageal cancer cells *in vitro* by negatively regulating VEGFA.

## Introduction

Esophageal cancer is a prevalent malignant tumor 1, 2. In recent years, it is found that esophageal cancer is not caused by a single factor, but involves multiple factors, including poor dietary habits, carcinogens, diseases and genetics 1-3. Excessive mould consumption, genetic predisposition, tobacco and alcohol addiction, and high intake of nitrosamines are risk factors that increase the susceptibility to esophageal cancer 4, 5. At present, men are more affected by esophageal cancer than women, with the ratio of 2-4: 1. Although esophageal cancer mainly affects people over 40 years, its incidence in the population younger than 40 years has increased 6, 7. With the continuous development of epidemiological research, the understanding of esophageal cancer has been gradually deepening. Nevertheless, its pathogenesis remains largely unclear 3, 8. It is increasingly important to find effective biomarkers for early diagnosis, treatment and prognosis prediction for esophageal cancer 9, 10.

Long non-coding RNAs (lncRNAs) are a group of noncoding RNAs in mammals that contain more than 200 nt in length 11, 12. LncRNAs play important roles in epigenetic, transcriptional, post-transcriptional and protein translation regulations 13, 14. Abnormally expressed lncRNAs have been identified as oncogenes or tumor suppressors, which are involved in various tumor cell behaviors 15, 16. Previous studies have shown that lncRNA TUSC8 is abundant in many types of cancers and participates in tumor progression 17, 18. However, its biological function in esophageal cancer remains unclear.

In this study, TUSC8 levels in esophageal cancer tissues and cell lines were detected. Subsequently, the *in vitro* influence of TUSC8 on esophageal cancer progression and the underlying mechanism were explored. Our findings provide novel ideas for clarifying the molecular mechanism of esophageal cancer, thus contributing to develop effective biomarkers for clinical screening and early diagnosis.

## Patients and methods

### Patients and esophageal cancer samples

Fresh cancer tissues and adjacent tissues were collected from 41 esophageal squamous cell carcinoma (ESCC) patients undergoing radical surgery, and placed in RNase-free EP tubes at -80 °C. Inclusion criteria were: ≥18 years old and pathologically confirmed as ESCC. The exclusion criterion is patient presented with other cancer types. This experiment was approved by the Medical Research Ethics Committee of Xinhua Hospital Affiliated to Shanghai Jiaotong University School of Medicine, and patients signed informed consent. Pathological classification and staging of ESCC were determined based on the criteria proposed by the Union for International Cancer Control (UICC). This study complied with the Helsinki Declaration.

### Cell lines and reagents

Human esophageal cancer cell lines (OE19, OE33, TE-1, KYSE30 and EC-109) and the normal esophageal epithelial cell line (HEEC) were purchased from American Type Culture Collection (ATCC) (Manassas, VA, USA). Cells were cultivated in Roswell Park Memorial Institute 1640 (RPMI 1640) (HyClone, South Logan, UT, USA) in a humidified incubator with 5% CO2 at 37 °C. 10% fetal bovine serum (FBS) (HyClone, South Logan, UT, USA), 100 U/mL penicillin and 100 μg/mL streptomycin were supplemented in RPMI 1640.

### Transfection

TUSC8 knockdown and overexpression plasmids, and their negative controls (100 nmol/L) were respectively transfected in cells using Lipofectamine^TM^ 2000 (Invitrogen, Grand Island, NY, USA). Transfection efficacy was examined at 48 h.

### Cell proliferation assay

2×10^3^ cells were implanted in each well of a 6-well plate and cultured for 1, 2, 3, or 4 days, where 10 μL of cell counting kit-8 (CCK-8) solution was added (TaKaRa, Japan). After 1-h culturing in the dark, the optical density at 490 nm was measured using a microplate reader.

### Colony formation assay

300 cells were implanted in each well of a 6-well plate and cultured for 10 days. Visible colonies were fixed in methanol for 10-15 days and stained using crystal violet. They were captured for counting.

### Transwell migration assay

Transwell chambers (Corning, NY, USA) were inserted in the 24-well plate, where 200 μL of suspension containing 1×10^5^ cells and 600 μL of RPMI 1640 with 20% FBS were respectively applied on the top and bottom. After 16 or 20 h cell culture, penetrating cells on the bottom were fixed using 4% paraformaldehyde solution for 20 min, air dried and dyed using crystal violet. Ten minutes later, cells were washed and captured for calculating migratory cells in 5 fields per sample.

### Quantitative real-time polymerase chain reaction (qRT-PCR)

TRIzol (Invitrogen, Grand Island, NY, USA) was used for isolating total RNAs, which were reversely transcribed. qRT-PCR system was prepared using the cDNA as the template and it was conducted using the GoTag Green Master Mix/Platinum SYBR SuperMix (TaKaRa, Japan). Three independent records were calculated by the formula: RQ=2^-∆∆Ct^, and the data were analyzed by the ABI Step One software (Applied Biosystems, Foster City, CA, USA). The following primers were used: TUSC8: 5'- GAUCAGCAUACACAAAUUA-3' (forward) and 5'-AGAAAGAUAUCAACAA-3' (reverse); VEGFA: 5'-TGTCTAATGCCCTGGAGCCT-3' (forward) and 5'- GCTTGTCACATCTGCAAGTACG-3' (reverse); GAPDH: 5'- CAACAGCCTCAAGATCATC-3' (forward) and 5'-ACCAGGAAATGAGCTTGAC-3' (reverse).

### Western blot

Cells were lysed in radioimmunoprecipitation assay (RIPA) (Beyotime, Shanghai, China) and the concentration of isolated protein was measured by bicinchoninic acid (BCA) method (Beyotime, Shanghai, China). Protein samples were separated by sodium dodecyl sulphate-polyacrylamide gel electrophoresis (SDS-PAGE), and transferred on polyvinylidene fluoride (PVDF) membranes (Millipore, Billerica, MA, USA). Subsequently, membranes were soaked in 5% skim milk for 2 hours. Primary antibodies (VEGFA, ab46154, Abcam) were applied for overnight incubation at 4 °C. On the next day, horse radish peroxidase (HRP)-labeled secondary antibodies were used for 2 h incubation. Band exposure was achieved by electrochemiluminescence (ECL) with glyceraldheyde 3-phosphate dehydrogenase (GAPDH, #2118, Cell Signaling Technology) as the internal reference.

### Dual-luciferase reporter assay

A sequence containing 300 base pairs upstream and downstream of the 3ʹ- untranslated region (3ʹ-UTR) conservative binding site of TUSC8 and the target gene VEGFA was synthesized, and amplified, respectively. PCR products were purified after amplification. The amplified sequence was double digested with pcDNA3.1 (+) - luciferase vector for acquiring TUSC8 luciferase vectors, which were co-transfected into cells with NC or pcDNA-VEGFA. Luciferase activity (Promega, Madison, WI, USA) was measured based on the standard procedures.

### Statistical analysis

Data analysis was performed using Statistical Product and Service Solutions (SPSS) 22.0 software (IBM, Armonk, NY, USA). Measurement data were expressed as mean ± standard deviation (x ± SD). Difference between two groups was analyzed using the Student's t-test. Comparisons among more than two groups were performed using ANOVA. The relationship between TUSC8 and clinical features in esophageal cancer patients was analyzed by Chi-square test. Kaplan-Meier method was used for survival analysis, and differences between curves were compared by Log-rank test. *p*<0.05 was considered statistically significant.

## Results

### The expression pattern of TUSC8 in esophageal cancer

**Figure 1:**
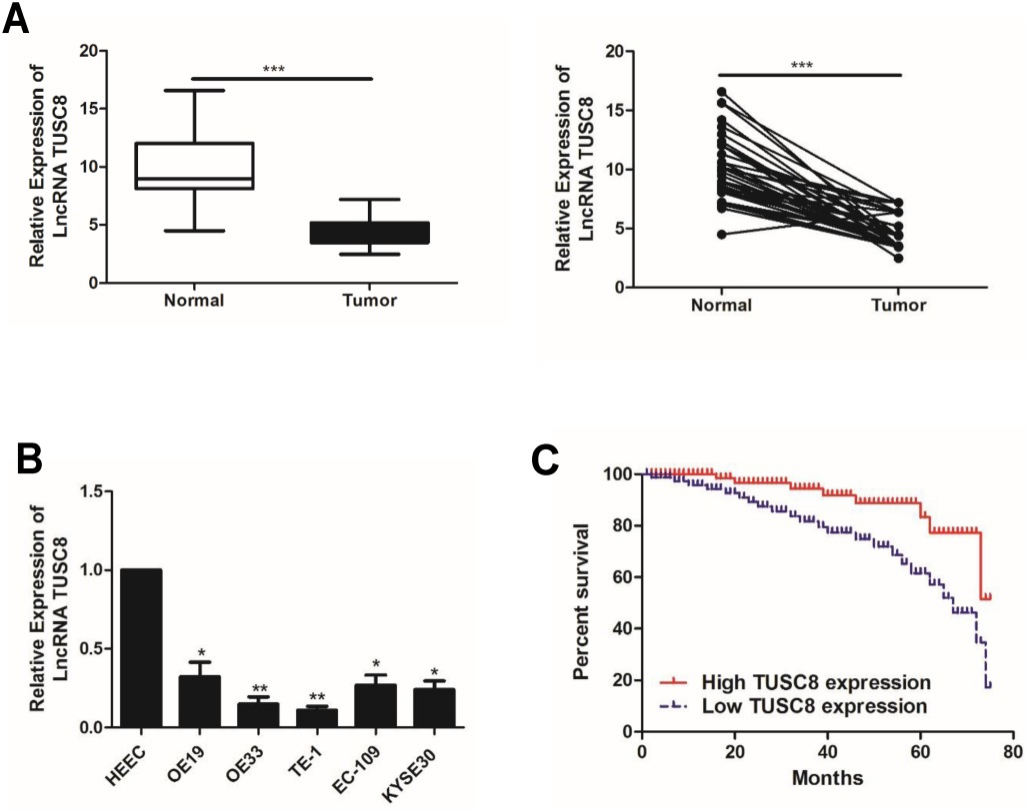
** The expression pattern of TUSC8 in esophageal cancer. A,** Differential expressions of TUSC8 in esophageal cancer tissues and normal ones detected by qRT-PCR. **B,** TUSC8 levels in esophageal cancer cell lines detected by qRT-PCR. **C,** Kaplan-Meier curves based on TUSC8 expressions in esophageal cancer patients. The prognosis was worse in the group with low TUSC8 expression than that in the group with high TUSC8 expression. Data were expressed as mean±SD. *p < 0.05, **p < 0.01, ***p < 0.001.

Compared with normal tissues, TUSC8 was lowly expressed in esophageal cancer tissues (Figure 1A). Consistently, the abundance of TUSC8 was downregulated in esophageal cancer cell lines compared with normal cells (Figure 1B). In particular, highly differentiated esophageal cancer cell lines OE19 and TE-1 cells were selected for subsequent experiments.

The statistical analysis on the relationship between TUSC8 and clinical features in esophageal cancer patients showed that TUSC8 was correlated to T stage and distant metastasis (*p*<0.05). However, it was unrelated to age, gender and lymph node metastasis (*p*>0.05) (Table 1). The Kaplan-Meier method was introduced to assess overall survival in esophageal cancer patients. The results of univariate analysis showed that low expression of TUSC8 was associated with poor prognosis in esophageal cancer (*p*<0.05, Figure 1C).

### TUSC8 suppresses the proliferative and migratory abilities in esophageal cancer cells

**Figure 2:**
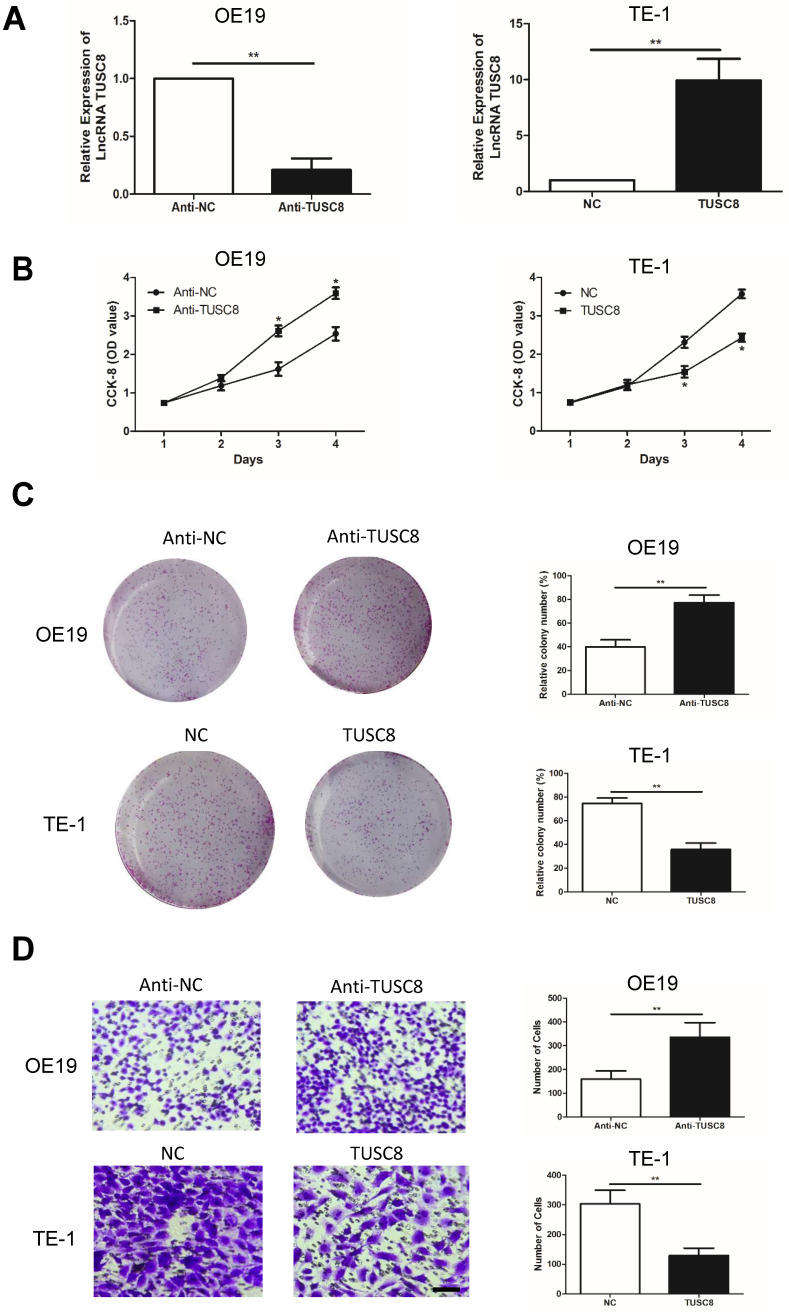
** TUSC8 suppressed the proliferative and migratory abilities of esophageal cancer cells. A,** Transfection efficacy of anti-TUSC8 and pcDNA-TUSC8 in OE19 and TE-1 cells detected by qRT-PCR, respectively. **B,** CCK-8 assay showed cell viability in OE19 and TE-1 cells regulated by TUSC8. **C,** Colony formation assay showed clonality in OE19 and TE-1 cells regulated by TUSC8 (Magnification: 40×). **D,** Transwell assay showed migration in OE19 and TE-1 cells regulated by TUSC8 (Magnification: 40×). Data were expressed as mean±SD. *p < 0.05, **p < 0.01.

To uncover the biological functions of TUSC8 in esophageal cancer cells, we intervened with the TUSC8 level in OE19 and TE-1 by transfection of anti-TUSC8 and pcDNA- TUSC8, respectively (Figure 2A). CCK-8 assay showed the viability was markedly enhanced in OE19 cells transfected with anti-TUSC8 than the control cells. Conversely, the viability in TE-1 cells overexpressing TUSC8 was significantly decreased (Figure 2B). Besides, colony number was increased in OE19 cells after knockdown of TUSC8 and it was reduced in TE-1 with TUSC8 overexpression (Figure 2C). Transwell assay revealed that the number of migratory cells was higher in OE19 cells transfected with anti-TUSC8 compared with those transfected with anti-NC. Overexpression of TUSC8 obtained the opposite trend in TE-1 cells (Figure 2D).

### VEGFA is a target gene of TUSC8

**Figure 3:**
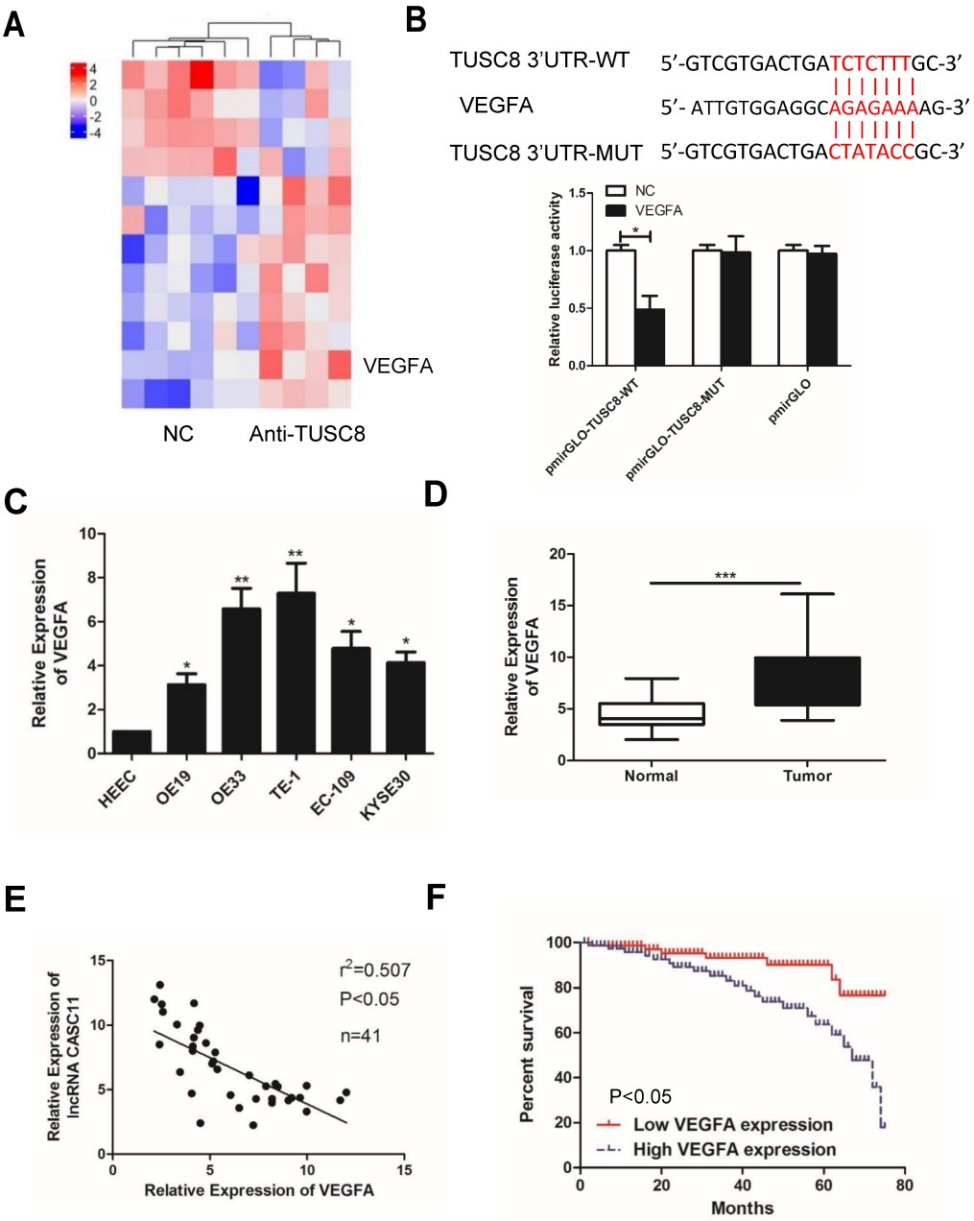
** VEGFA was a target gene of TUSC8. A,** High-throughput sequencing of transcriptome suggested that VEGFA expression was significantly changed after knockdown of TUSC8. **B,** Dual-luciferase reporter assay confirmed the binding between TUSC8 and VEGFA. **C,** VEGFA levels in esophageal cancer cell lines detected by qRT-PCR. **D,** Differential expressions of VEGFA in esophageal cancer tissues and normal ones detected by qRT-PCR. **E,** TUSC8 level was negatively correlated to VEGFA level in esophageal cancer tissues. **F,** Kaplan-Meier curves based on VEGFA expressions in esophageal cancer patients. The prognosis was worse in high VEGFA expression group than that in low VEGFA expression group. Data were expressed as mean±SD. *p < 0.05, **p < 0.01, ***p < 0.001.

To explore how TUSC8 inhibits the malignant progression of esophageal cancer, high-throughput sequencing of transcriptome was conducted. It was shown that the expression level of VEGFA was pronouncedly changed after knockdown of TUSC8 (Figure 3A). Subsequently, the dual-luciferase reporter assay confirmed that TUSC8 could bind VEGFA (Figure 3B). VEGFA was upregulated in esophageal cancer cell lines and tissues (Figure 3C, 3D). Notably, VEGFA was negatively correlated to TUSC8 level in esophageal cancer tissues (Figure 3E). Kaplan-Meier curves demonstrated that high level of VEGFA was closely linked to poor prognosis in esophageal cancer (*p*<0.05, Figure 3F).

### TUSC8 promotes the aggravation of esophageal cancer via negatively regulating VEGFA

**Figure 4:**
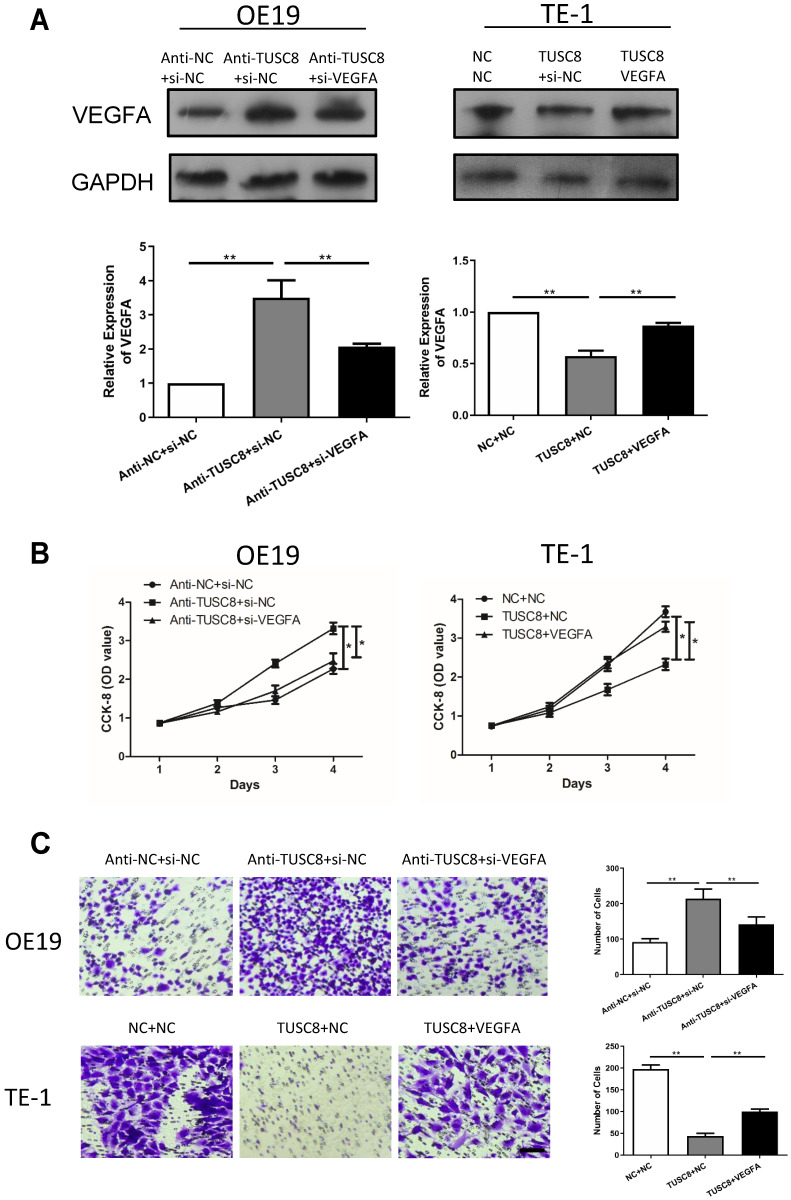
** TUSC8 promoted the aggravation of esophageal cancer via negatively regulating VEGFA. A,** The transfection efficacy of si-VEGFA and pcDNA-VEGFA in OE19 and TE-1 cells with intervened TUSC8 level detected by Western blot, respectively. **B,** CCK-8 assay showed cell viability in OE19 and TE-1 cells regulated by TUSC8 and VEGFA. **C,** Transwell assay showed migration in OE19 and TE-1 cells regulated by TUSC8 and VEGFA (Magnification: 40×). Data were expressed as mean±SD. *p < 0.05, **p < 0.01.

Rescue experiments were conducted to elucidate the involvement of VEGFA in esophageal cancer progression regulated by TUSC8. Transfection efficacy of si- VEGFA and pcDNA-VEGFA was examined in OE19 and TE-1 cells intervened with TUSC8 level, respectively (Figure 4A). Silencing of VEGFA reversed the enhanced effects of TUSC8 knockdown on the viability and migration in OE19 cells. On the contrary, overexpression of VEGFA restored the proliferative and migratory abilities inhibited by TUSC8 overexpression in TE-1 cells (Figure 4B, 4C).

## Discussion

Esophageal cancer is a highly heterogeneous malignant tumor with high morbidity and mortality worldwide 1-3. Due to the differences in geographical location and ethnicity, histological subtypes of esophageal cancer are diverse. In China, ESCC is the main subtype of esophageal cancer, which is highly prevalent in Hebei, Shanxi, and Henan province 4-6. The occurrence of ESCC is a multifactorial process 1, 2. The low detective rate of early stage ESCC and its poor prognosis should be well concerned 2, 3, 6, 7. It is urgent to seek for esophageal cancer biomarkers for clinical application 9, 10.

The activation of oncogenes and loss of function of tumor suppressors are responsible for carcinogenesis 19, 20. LncRNAs are closely involved in tumor progression as vital regulators 11, 12. Notably, the tissue and time-specificity of lncRNAs are much more pronounced than those of mRNAs 13, 14. LncRNAs have been discovered to be closely linked to tumor progression, metastasis, recurrence and prognosis. A previous study has shown that lncRNA H19 is upregulated in esophageal cancer, which induces cell cycle blockage in G1 phase and stimulates proliferative ability by regulating miR-675-5p, serving as a prognostic indicator 21. LncRNA MALAT1 is highly abundant in esophageal cancer tissues, and positively correlated to clinical stage, tumor size and lymphatic metastasis incidence. Knockdown of MALAT1 weakens the proliferative and metastatic ability in esophageal cancer cells, and induces cell apoptosis by blocking cell cycle progression in G2/M phase *via* phosphorylating the ATM-CHK2 signaling 22. TUSC8 is a newly discovered lncRNA and exerts its anti-carcinogenic role by targeting downstream genes, such as MYLIP and PTEN 17, 18. Thus, decreased TUSC8 expression creates a favorable microenvironment for tumor cell growth. Consistently, our findings uncovered downregulated TUSC8 expression in esophageal cancer tissues and cell lines. By intervening TUSC8 level in OE19 and TE-1 cells, our data revealed that TUSC8 attenuated the proliferation and migration in esophageal cancer cells, indicating that TUSC8 acts as a tumor suppressor in esophageal cancer.

To explore how TUSC8 inhibits the malignant progression of esophageal cancer, high-throughput sequencing of transcriptome was conducted. The result showed that the expression level of VEGFA was pronouncedly changed after knockdown of TUSC8. VEGFA is a cytokine that regulates vascular development and new blood vessel formation 23. VEGFA has been reported to be expressed in many cancers and its high expression is associated with poor prognosis 24. VEGFA is suggested to be a key regulator in tumorigenesis and cancer progression 25. In the present study, the binding relationship between TUSC8 and VEGFA was further confirmed, and they displayed a negative correlation in esophageal cancer tissues. Importantly, VEGFA was able to reverse the regulatory effects of TUSC8 on esophageal cancer cell behaviors. Our findings reveal a co-regulation of TUSC8 and VEGFA in esophageal cancer progression, and are conductive to clinical diagnosis and treatment of esophageal cancer.

## Conclusion

LncRNA TUSC8 is downregulated in esophageal cancer tissues and cell lines. It inhibits proliferative and migratory abilities in esophageal cancer *in vitro* by negatively regulating VEGFA.

## Figures and Tables

**Table 1:** Association of LncRNA TUSC8 expression with clinicopathologic characteristics of esophageal cancer

Parameters	Number of cases	LncRNA TUSC8 expression		*p*-value
		Low (%)	High (%)	
**Age (years)**				0.218
<60	15	11	4	
≥60	26	14	12	
**Gender**				0.262
Male	20	13	7	
Female	21	10	11	
T stage				0.006
T1-T2	25	20	5	
T3-T4	16	6	10	
**Lymph node metastasis**				0.083
No	26	20	6	
Yes	15	7	7	
**Distance metastasis**				0.047
No	31	23	8	
Yes	10	4	6	
